# Effect of two-step mock-up methods on the trueness of complete-arch trial restorations: an in vitro study

**DOI:** 10.1186/s12903-025-06978-0

**Published:** 2025-10-10

**Authors:** Chunxiao Jin, Weiwei Huang, Liya Ma, Yueli Zhou, Mengxun Li, Yake Wang, Cui Huang

**Affiliations:** 1 State Key Laboratory of Oral & Maxillofacial Reconstruction and Regeneration, Key Laboratory of Oral Biomedicine Ministry of Education, Hubei Key Laboratory of Stomatology, School & Hospital of Stomatology, Wuhan University, Wuhan, 430079 China; 2 Center for Digital Technology and Telemedicine at Optics Valley Branch, School & Hospital of Stomatology, Wuhan University, Wuhan, 430079 China

**Keywords:** Mock-up, Waxing, Trial restoration, Trueness, Silicone matrix index, Full-mouth rehabilitation

## Abstract

**Background:**

To assess the trueness of trial restorations fabricated using the two-step mock-up methods with varying distributions of hard tissue stop areas in the complete-arch mock-up process and to compare these outcomes with the one-step method.

**Methods:**

Complete-arch digital diagnostic waxings were designed on a worn maxillary cast. The original waxings were modified into four intermediate versions, each retaining a different set of original worn teeth: bilateral molars, molars and incisors, lateral incisors along with canines and molars, or alternating teeth. One complete-arch waxing cast, four intermediate waxing casts and 25 original worn casts were 3D printed, and silicone indices were prepared. Trial restorations were made by injecting bis-acrylic resin into the silicone indices and curing under pressure. Five groups were formed, one-step (Group 1) and four two-step methods (Groups 2–5). Root mean square (RMS) was applied for 3D analysis (global and tooth levels). Point-to-point measurements were used for 2D occlusal surfaces. One-way ANOVA with post-hoc tests was performed to assess intergroup differences.

**Results:**

Significant differences in overall trueness were found among the five groups (F (4,20) = 92.61, *P* < 0.001). Group 1 (one-step) showed the largest mean RMS deviation (0.31 ± 0.02 mm) while Group 5 (alternating teeth) had the smallest (0.15 ± 0.01 mm), not significantly different from Group 4 (0.17 ± 0.01 mm). Tooth-level 3D analyses revealed a similar pattern, with the largest deviations observed at premolars (0.34 ± 0.07 mm) and canines (0.60 ± 0.09 mm) in Group 1, both significantly greater than Groups 4 and 5 (< 0.17 mm, all *P* < 0.001). Occlusal surface 2D analyses showed statistically significant differences among groups (all *P* < 0.001), with Group 1 reaching 0.58 ± 0.12 mm at premolars compared with 0.16 ± 0.09 mm in Group 5.

**Conclusions:**

The two-step methods enhance the trueness of complete-arch trial restorations compared to the one-step method, with increased supporting teeth reducing overall deviations.

## Background

Tooth wear, a progressive condition recognized by the World Health Organization as part of aging, significantly influences chewing function and quality of life [1–3]. Its global prevalence in permanent teeth ranges from 26.9% to 90.0%, with severe cases affecting 25% of young adults and 17% of elderly individuals [4, 5]. Approximately 10% of patients require restorative interventions involving vertical dimension increase and occlusal reconstruction [6–9].

Trial restorations serve as critical diagnostic tools in these rehabilitations, enabling evaluation of occlusion, aesthetics, and minimally invasive preparation [10–12]. Clinically, both analog (e.g., silicone matrix-index) and digital (milled/printed) fabrication methods are routinely employed. While digital workflows offer efficiency advantages, including reduced chairside time and enhanced mechanical properties [13–15], analog techniques are widely used due to their procedural simplicity and chairside adaptability [10, 16, 17]. This continued reliance on analog methods has motivated ongoing refinement of their accuracy.

Strategic material selection and appropriate silicone modifications can enhance the trueness of trial restorations. Koh et al. and Taha et al. demonstrated comparable outcomes between digital trial restorations and high-hardness silicone putty techniques [11, 15]. Li et al. achieved improved thickness control of trial restorations through equigingival silicone matrices with palatal notches [10]. Moldovani et al. found that bis-acryl performed better than acrylic resins in analog workflows [18]. Despite these advances, current evidence derives predominantly from anterior or segmental trial restorations. A critical knowledge gap persists: no studies have quantified the accuracy of complete-arch trial restorations, nor developed accuracy-optimization strategies for these trial restorations. To address this dual limitation, we propose a two-step mock-up method incorporating intermediate casts with original teeth as hard-tissue stops [19, 20].

This study aimed to evaluate the trueness of complete-arch trial restorations fabricated via two-step mock-up method with varying stop distributions versus conventional one-step techniques. The null hypothesis stated that there would be no differences in trueness between one-step and two-step mock-up methods with different stop distributions.

## Methods

### Preparation of waxing casts

**Fig. 1:**
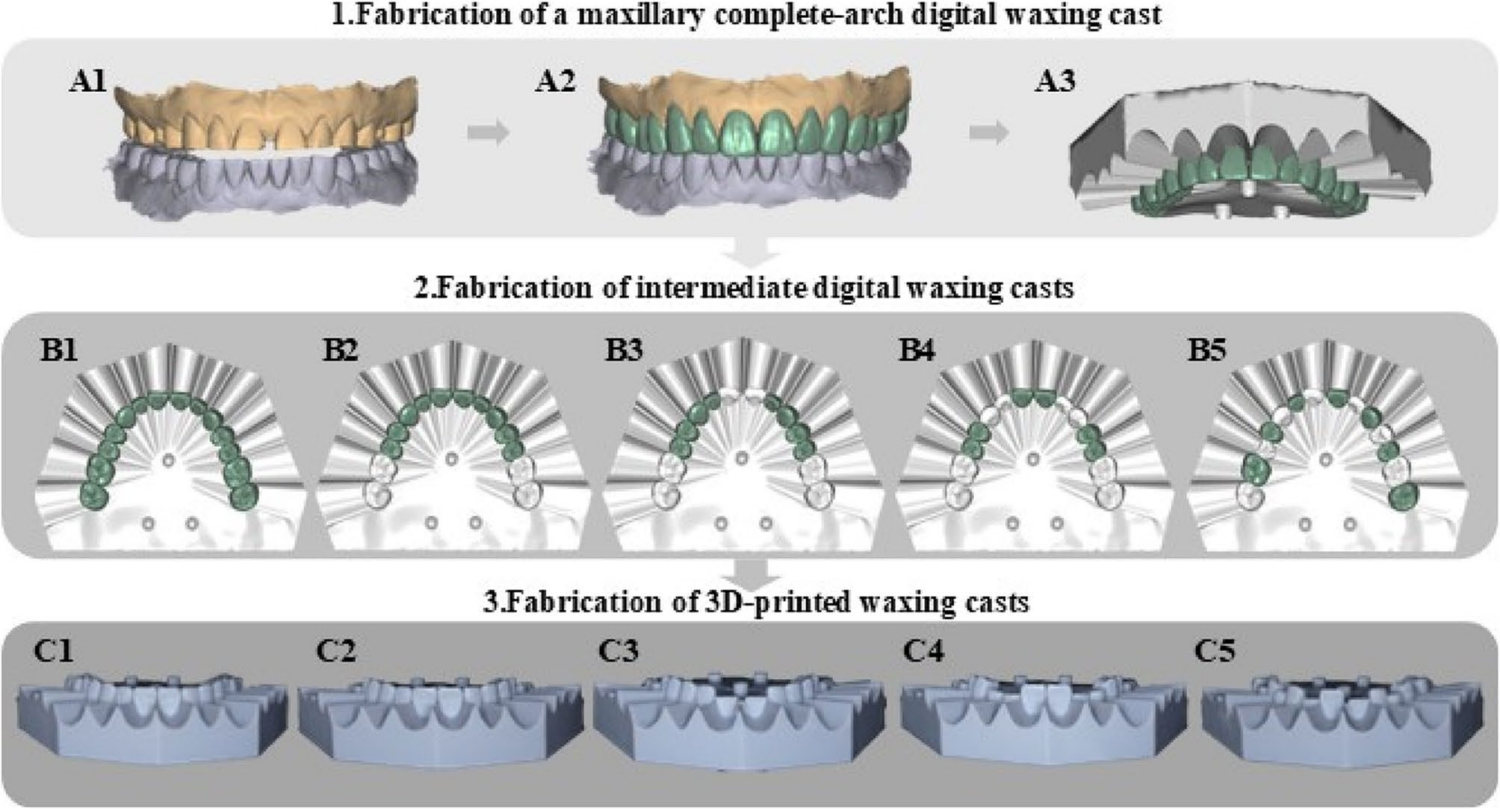
Fabrication of composite diagnostic waxing casts. (A1) Digital worn dentition cast. (A2) Digital waxing. (A3) Silicone matrices gingival margin stop guide. (B1-C1) Complete-arch waxing cast without original worn teeth. (B2-C2) Waxing cast retained original bilateral molars. (B3-C3) Waxing cast retained original bilateral molars and incisors. (B4-C4) Waxing cast retained original bilateral molars, lateral incisors and canines. (B5-C5) Waxing cast retained original alternating teeth of the worn dentition cast. Green teeth represent diagnostic waxings, and silver teeth represent original worn teeth

Digital casts of the maxillary and mandibular arches of a patient with severely worn dentition at an increased vertical dimension of occlusion were obtained. Digital diagnostic waxings were designed using dental CAD software (DentalCad^®^, Exocad, Darmstadt, Germany) based on the patient’s increased maxillomandibular relationship and esthetic preferences. The complete-arch waxing cast was imported into CAD software (Meshmixer, Autodesk, San Rafael, USA), where the gingival margin areas on both the labial and lingual sides were selected and extended to create a silicone matrix gingival margin stop structure. This structure was then combined with the complete-arch maxillary waxing cast (Fig. 1). The composite waxing cast was used directly to fabricate trial restorations for the one-step mock-up method, designated as Group 1, and also served as the waxing cast for the second step of the two-step mock-up process.

Afterward, selected waxings were removed from specific teeth to create different first intermediate waxing casts for the two-step mock-up processes. Based on the positions of the original abutment teeth retained in the first intermediate waxing cast, four digital waxing casts were generated, each corresponding to a different experimental group (Fig. 1B1-B5):

Group 2: Waxing cast retained the original bilateral molars.

Group 3: Waxing cast retained the original bilateral molars and incisors.

Group 4: Waxing cast retained the original bilateral molars, lateral incisors, and canines.

Group 5: Waxing cast retained alternating teeth.

The sample size was determined by a priori power analysis using G*Power (version 3.1; Heinrich Heine University Düsseldorf, Germany). Based on an effect size of 0.8, α = 0.05, and 80% power, a sample size of *n* = 5 per group was required for this exploratory in vitro study.

### Fabrication of silicone indices

**Fig. 2:**
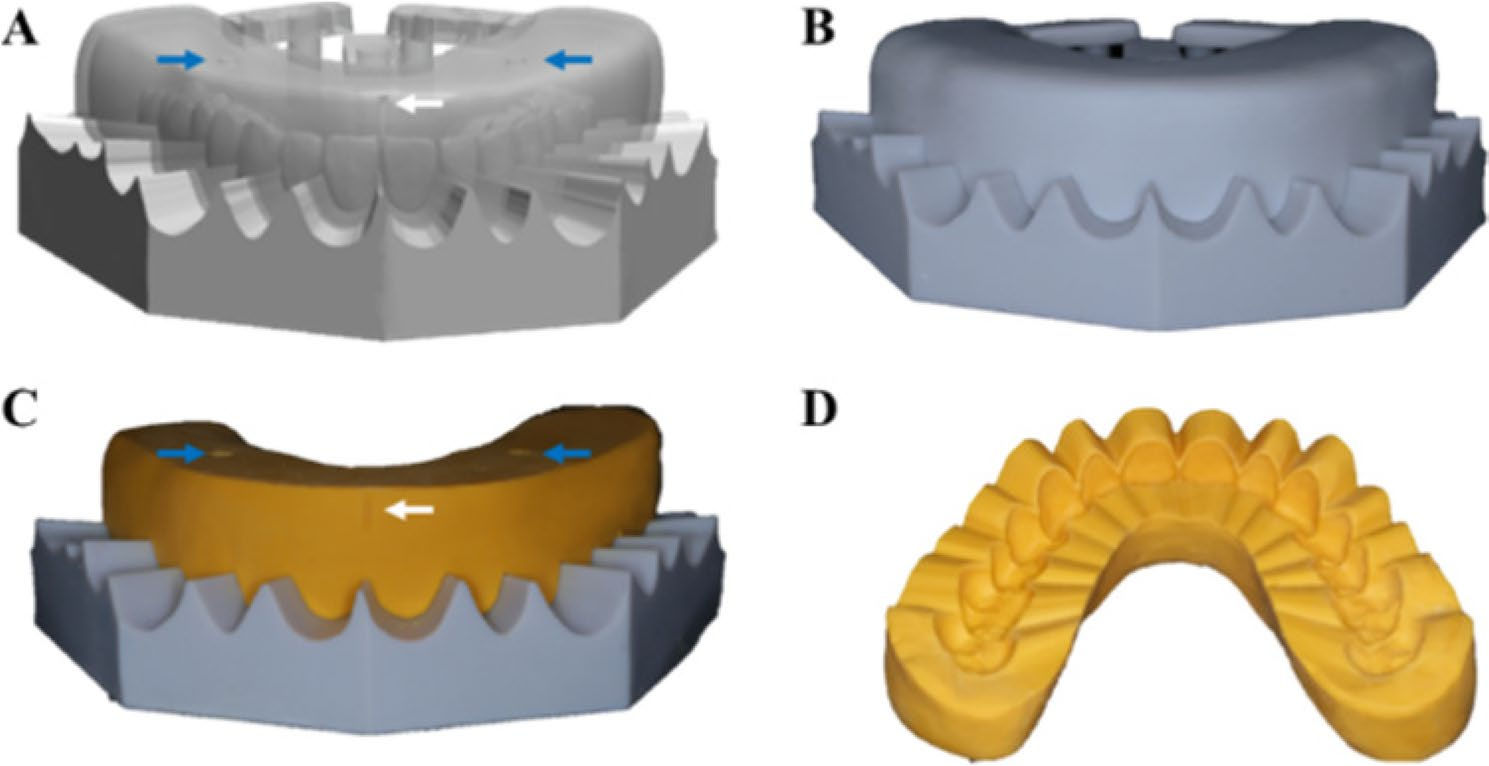
Fabrication procedure of silicone index. **A** Digital silicone index mold. **B** 3D-printed silicone index mold. **C** Outer surface of the silicone index. **D** Inner surface of the silicone index

A mold was designed to fabricate the silicone matrix indices, ensuring a consistent thickness of 8 mm and a uniform shape. Two weight indicator points were incorporated into the bilateral premolar areas to standardize the application of external forces during the process (Fig. 2).

All subsequent procedures were performed under controlled laboratory conditions (temperature: 23 ± 1 °C, relative humidity: 50 ± 5%) by one trained experienced operator to minimize variability. Five waxing casts and the mold were 3D-printed using dimethacrylate acrylic resin (Model HP UV Grey, HeyGears, Guangzhou, China) with a 3D printer (UltraCraft A3D, HeyGears, Guangzhou, China) (Fig. 1C1-C5). For the initial digital waxing process of the two-step method, five silicone matrix indices (Express XT Putty Soft, 3 M ESPE, Seefeld, Germany) were fabricated for each intermediate waxing cast. Additionally, 25 silicone matrix indices were prepared for fabricating a complete-arch trial restoration. Of these, five indices were allocated for the one-step method, while the remaining 20 were used in the second step of the two-step method.

### Mock-up procedures

**Fig. 3:**
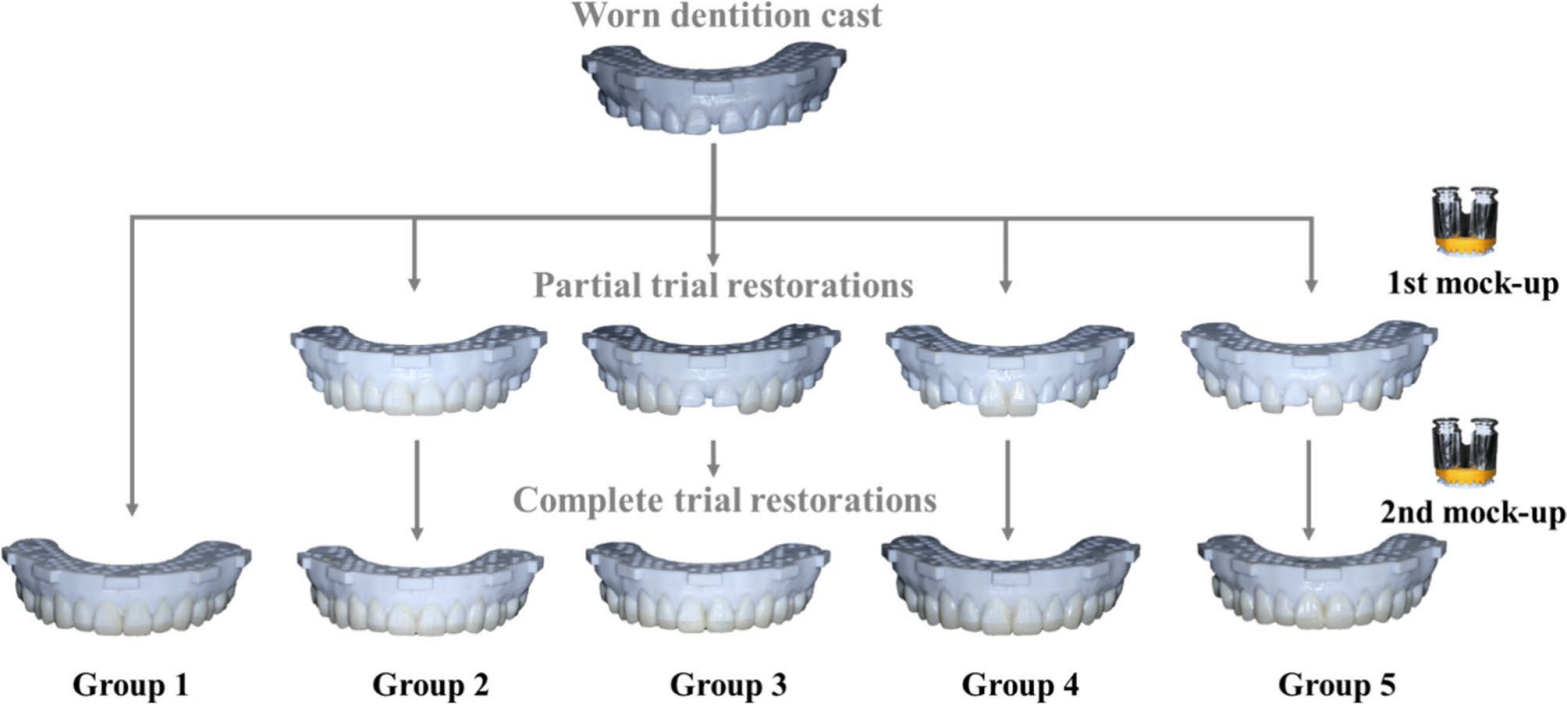
Fabrication procedure of trial restorations using one-step mock-up method (Group 1) and two-step mock-up methods (Group 2–5)

In the one-step method, trial restorations were fabricated entirely in a single process. For the two-step methods, the first step created partial trial restorations using the initial silicone index, and the second step completed the remaining teeth with the second silicone index (Fig. 3). Bis-acrylic resin (Structur 2 SC, color A2, VOCO GmbH, Cuxhaven, Germany) was injected evenly into the silicone matrix index using a mixing gun within 20 s. The injection progressed from the leftmost to the rightmost tooth, filling two-thirds of each tooth’s space. The silicone index loaded with resin was immediately seated onto the worn cast within 10 s. Two 500 g weights were applied to the bilateral premolar areas to ensure consistent pressure during resin polymerization, with the setting time maintained at 10 min for complete curing. Afterward, the silicone index and excess resin were removed. The trial restorations were then stored under controlled laboratory conditions (temperature: 23 ± 1 °C, relative humidity: 50 ± 5%) for 24 h before being scanned using a pre-calibrated laboratory scanner (E4, 3 Shape, Copenhagen, Denmark). The scan data were exported as standard tessellation language (STL) files. Both the scanned trial restoration STL files and the designed digital waxing cast STL files were imported into a software program (Geomagic Control X 2021, 3D Systems, Rock Hill, USA). The files were aligned using the “Reference Best Fit Alignment” command, with the base section serving as the common reference area.

### Data analysis

**Fig. 4:**
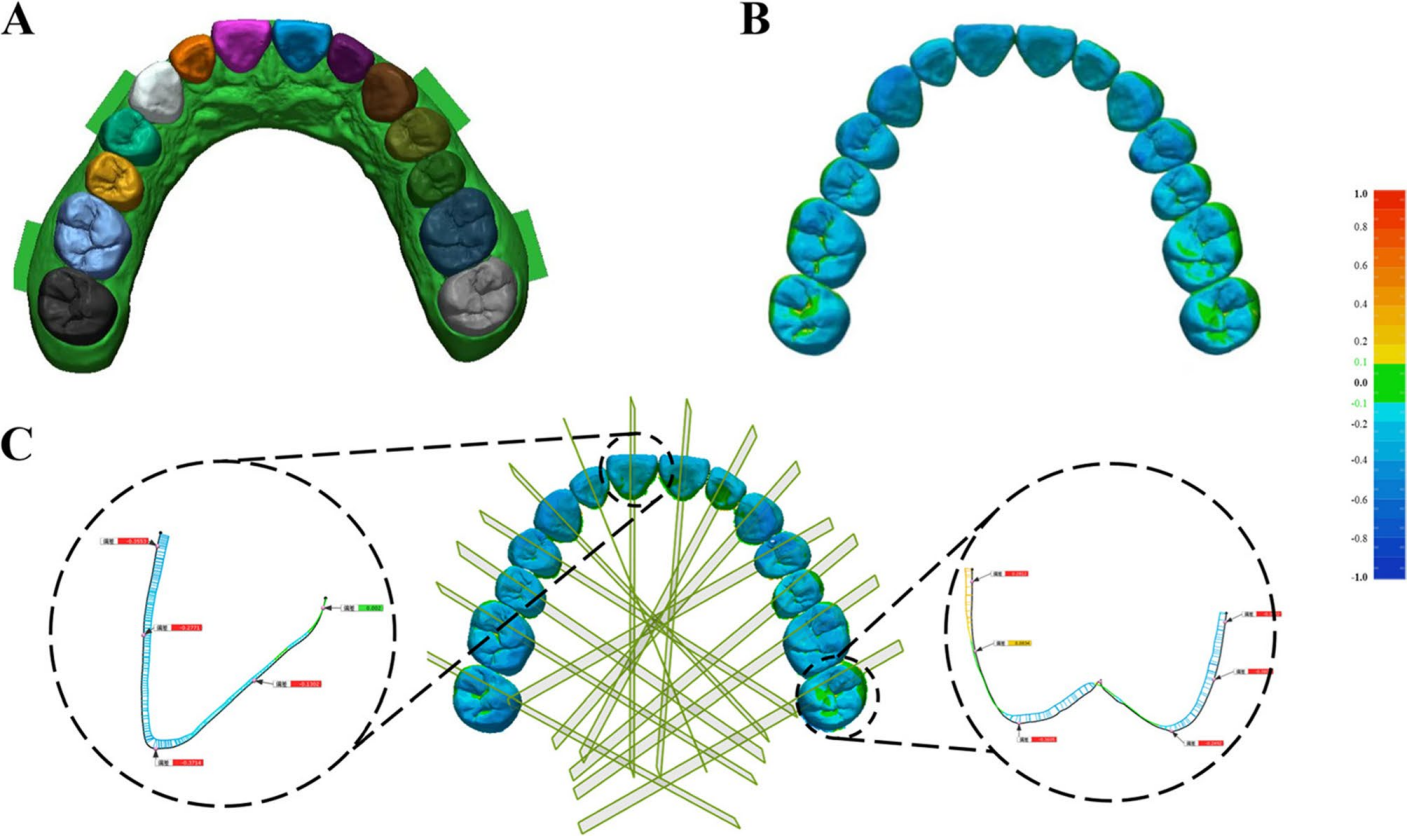
Deviation analysis procedure. **A** Tooth segmentation.** B** RMS for the complete-arch and each tooth position. **C** Measurement planes, perpendicular to the occlusal plane and aligned with the long axis of each trial restoration, were created to measure deviations at specific points: cervical, middle of the buccal and lingual surfaces, and the middle of incisal edges or cusp tips on the occlusal surface

The 3D deviations were analyzed using five independent specimens per group (*N* = 5), with each specimen scanned once under standardized conditions. For each scan, complete-arch and each tooth deviations were quantified as root mean square (RMS) (Fig. 4) [21], calculated using: $\:\text{RMS}=\sqrt{\frac{1}{n}{\sum\:}_{i=1}^{n}{\left({X}_{\text{ref},i}-{X}_{\text{scan},i}\right)}^{2}}$, where $\:{X}_{\text{ref},i}$ represents the reference data point at index i, $\:{X}_{\text{scan},i}$ denotes the scanned data point at index i, and *n* is the total number of measurement points in the analyzed region. Subsequently, a measurement section was created along the long axis of each trial restoration. The 2D deviations for each tooth were measured at the buccolingual cervical, middle, and incisal/occlusal locations along the section. For posterior teeth, the measurement section passed through both the buccal and lingual cusps. The average deviation of two cusps was used to represent the deviation on the occlusal surface of the tooth. The data for each tooth position were calculated as the average measurements of the corresponding bilateral homonymous teeth.

### Statistical analysis

For each experimental group, the mean ± standard deviation was computed based on data from five specimens. The Shapiro–Wilk test was used to assess data normality, and results confirmed that the data conformed to a normal distribution (*P* > 0.05). One-way analysis of variance (ANOVA) was then applied to further analyze variables with potential differences. For post-hoc comparisons, Tukey’s test was utilized for groups with homogeneous variances, whereas Tamhane’s T2 test was adopted for groups with heterogeneous variances. The statistical significance threshold was set at α = 0.05.

## Results

Significant differences in overall trueness were observed among the five experimental groups (F (4,20) = 92.61, *P* < 0.001, η² = 0.949), as detailed in Table 1. Group 1 (conventional one-step method) exhibited the largest mean RMS deviation (0.30 ± 0.02 mm), indicative of the poorest trueness. In contrast, Groups 4 and 5 (two-step methods) demonstrated the highest trueness, with the smallest deviations found in Group 5 (0.15 ± 0.01 mm). No significant difference was observed between these two groups (*P* > 0.05).

**Table 1:** Overall trueness across all experimental groups

	Mean	Standard Deviation	Minimum	Median	Maximum	95% Confidence Interval
Group1	0.30^A^	0.02	0.28	0.30	0.34	0.25–0.34
Group2	0.26^B^	0.00	0.25	0.26	0.26	0.25–0.26
Group3	0.21^C^	0.02	0.19	0.22	0.23	0.19–0.23
Group4	0.17^D^	0.01	0.15	0.17	0.19	0.15–0.19
Group5	0.15^D^	0.01	0.13	0.15	0.16	0.14–0.16
F (4,20)	92.61					
η²	0.949					
*P*	<0.001					

Different uppercase letters indicate significant differences among groups within the same column (*P* < 0.05).

**Fig. 5:**
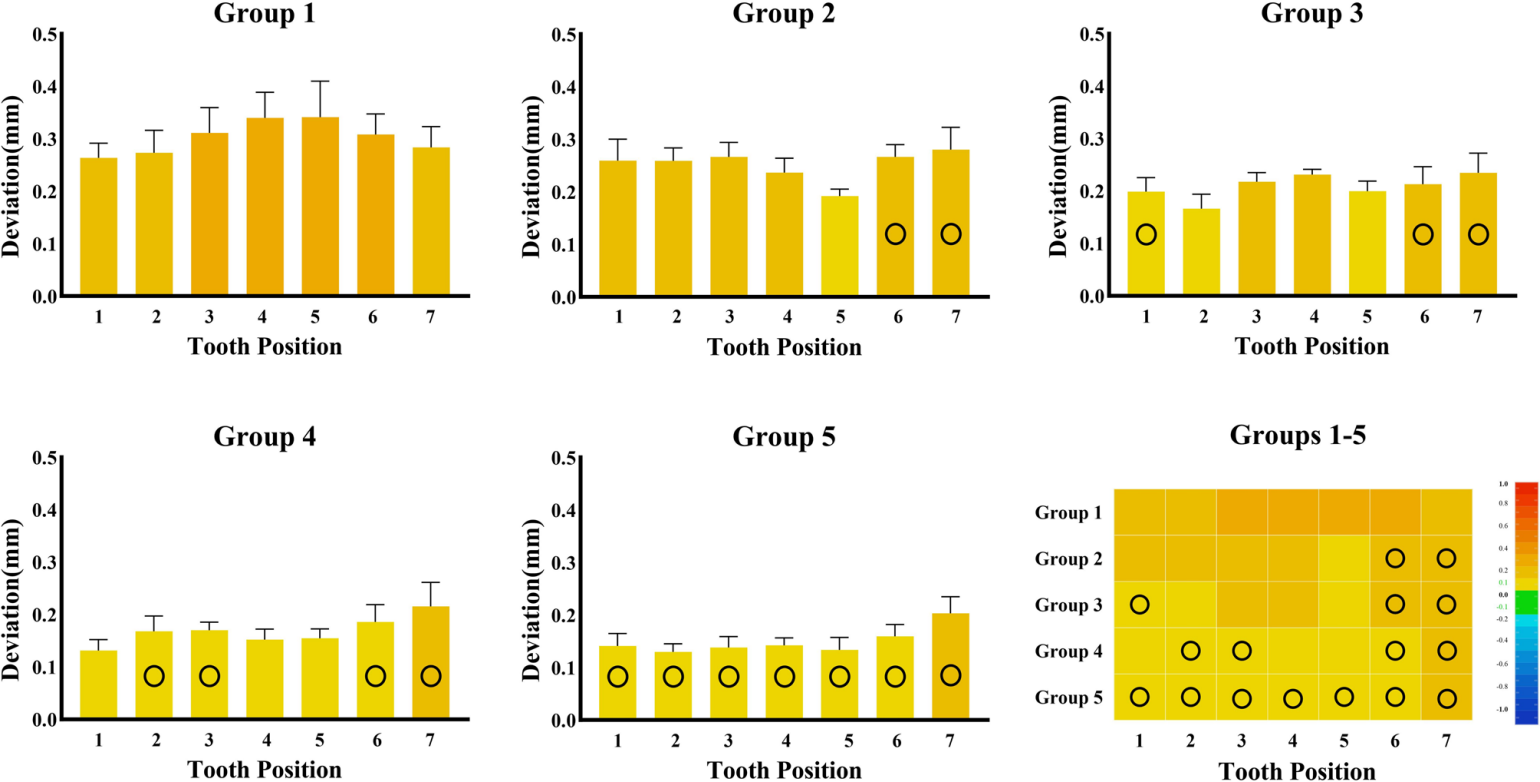
Bar charts and heat maps depicting the RMS values at each tooth position among groups. The black circles denote the original worn teeth that were retained in the first step of the two-step method. Yellow regions represent deviations greater than + 0.1 mm, where colors gradually approaching red indicate larger deviations

Tooth-level analysis revealed distinct patterns of deviation across the dental arch (Table 2). Figure 5 presents the RMS differences for each tooth in a bar chart format. A one-way ANOVA for each tooth position showed significant differences among groups (all *P* < 0.001), with large effect sizes (η² ranging from 0.772 to 0.878 for all teeth except second molars). In Group 1, premolars exhibited the highest deviations (0.34 ± 0.05 mm), significantly exceeding those of incisors (0.26 ± 0.03 mm, *P* < 0.001). As supporting teeth were incorporated in the two-step methods, deviations decreased in teeth located adjacent to hard stops (indicated by circles in Fig. 7). In Group 2, the smallest deviations occurred at the second premolars (0.19 ± 0.01 mm), which were positioned beside the hard stops located on the first molars. Similarly, in Group 3, minimal deviations were observed at the lateral incisors (0.17 ± 0.03 mm), located adjacent to the hard stops on the central incisors. Groups 4 and 5, which featured the most distributed support, exhibited the lowest deviations across all tooth positions, with the greatest improvements occurring in posterior regions.

**Table 2:** Comparison of RMS values at each tooth position

	Tooth position							Statistics	
	Central incisors	Lateral incisors	Canines	First premolars	Second premolars	First molars	Second molars	F (6.63) *P*	η² 95% CI
Group 1	0.26 ± 0.03^Aa^	0.27 ± 0.04^Aa^	0.31 ± 0.05^Aab^	0.34 ± 0.05^Ab^	0.34 ± 0.07^Ab^	0.31 ± 0.04^Aab^	0.28 ± 0.04^Aab^	4 0.378 < 0.001	0.295 0.480–0.543
Group 2	0.26 ± 0.04^Aa^	0.26 ± 0.02^Aa^	0.27 ± 0.03^Aa^	0.24 ± 0.03^Ba^	0.19 ± 0.01^Bb^	0.27 ± 0.02^Ba^	0.28 ± 0.04^Aa^	9.539 < 0.001	0.481 0.398–0.455
Group 3	0.2 ± 0.03^Bab^	0.17 ± 0.03^BCa^	0.22 ± 0.02^Bbc^	0.23 ± 0.01^Bb^	0.2 ± 0.02^Bac^	0.21 ± 0.03^Cab^	0.23 ± 0.04^ABbc^	7.949 < 0.001	0.427 0.334–0.364
Group 4	0.13 ± 0.02^Ca^	0.17 ± 0.03^Babc^	0.17 ± 0.02^Cbc^	0.15 ± 0.02^Cab^	0.15 ± 0.02^Cab^	0.19 ± 0.03^CDbc^	0.22 ± 0.05^Bc^	9.281 < 0.001	0.478 0.209–0.243
Group 5	0.14 ± 0.02^Ca^	0.13 ± 0.02^Ca^	0.14 ± 0.02^Da^	0.14 ± 0.01^Ca^	0.13 ± 0.02^Ca^	0.16 ± 0.02^Da^	0.2 ± 0.03^Bb^	12.79 0.005	0.549 0.123–0.160
F (4,45)	47.68	46.15	60.47	82.05	53.54	38.07	8.62		
*P*	< 0.001	< 0.001	< 0.001	< 0.001	< 0.001	< 0.001	< 0.001		
η²	0.81	0.803	0.845	0.878	0.824	0.772	0.432		
95% CI	0.181–0.217	0.181–0.217	0.201–0.240	0.199–0.242	0.181–0.227	0.209–0.244	0.230–0.258		

CI, confidence interval. Different uppercase letters indicate significant differences among groups within the same column (*P* < 0.05). Different lowercase letters indicate significant differences among groups within the same row (*P* < 0.05).

**Fig. 6:**
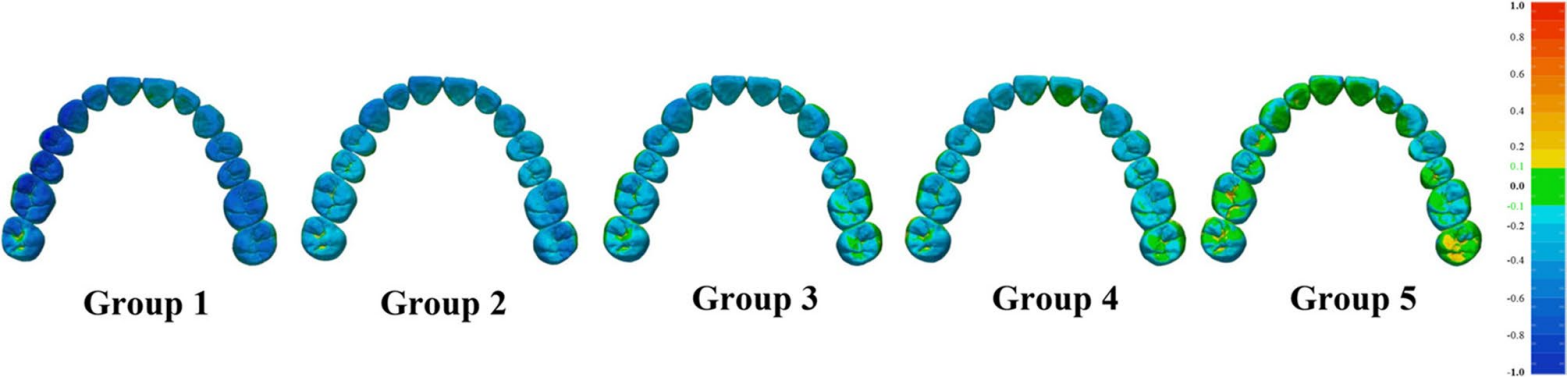
Graphical illustrations of fitting comparisons across all subgroups. Green areas represent deviations within ± 0.1 mm, yellow areas represent positive deviations above the reference (> + 0.1 mm), and blue areas represent negative deviations below the reference (< − 0.1 mm), with deeper colors indicating larger deviations

**Fig. 7:**
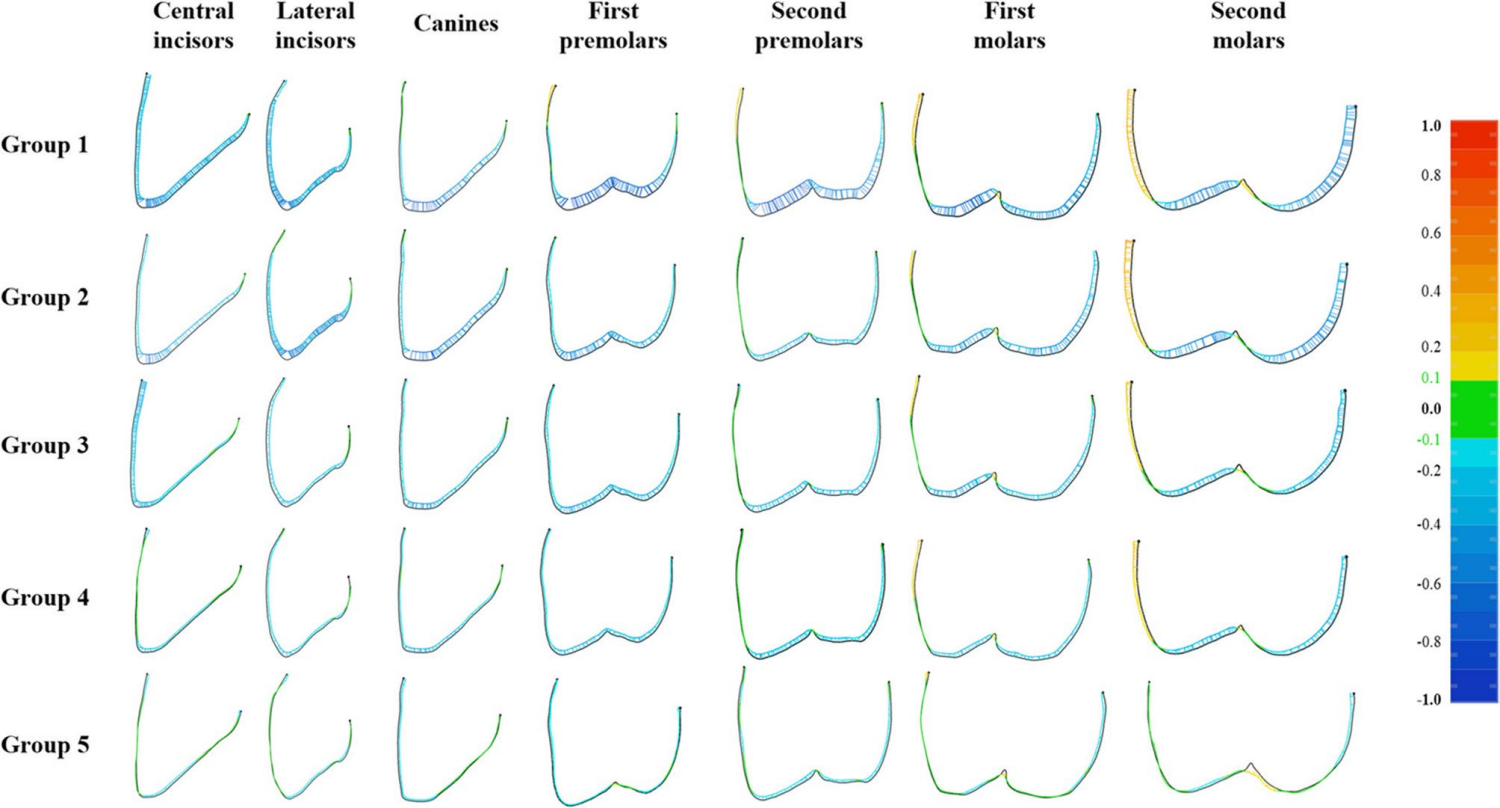
2D cross-sectional images of the right maxillary teeth. Green areas represent deviations within ± 0.1 mm, yellow areas represent positive deviations above the reference (> + 0.1 mm), and blue areas represent negative deviations below the reference (< − 0.1 mm), with deeper colors indicating larger deviations

Figure 6 displays color maps visualizing the 3D deviations between the test and reference casts from an occlusal view. Figure 7 presents 2D linear profiles derived from sagittal cross-sections of the right maxillary teeth. In both figures, green areas indicate deviations within ± 0.1 mm, yellow areas represent positive deviations (test surface above reference), and blue areas indicate negative deviations (test surface below reference).

**Fig. 8:**
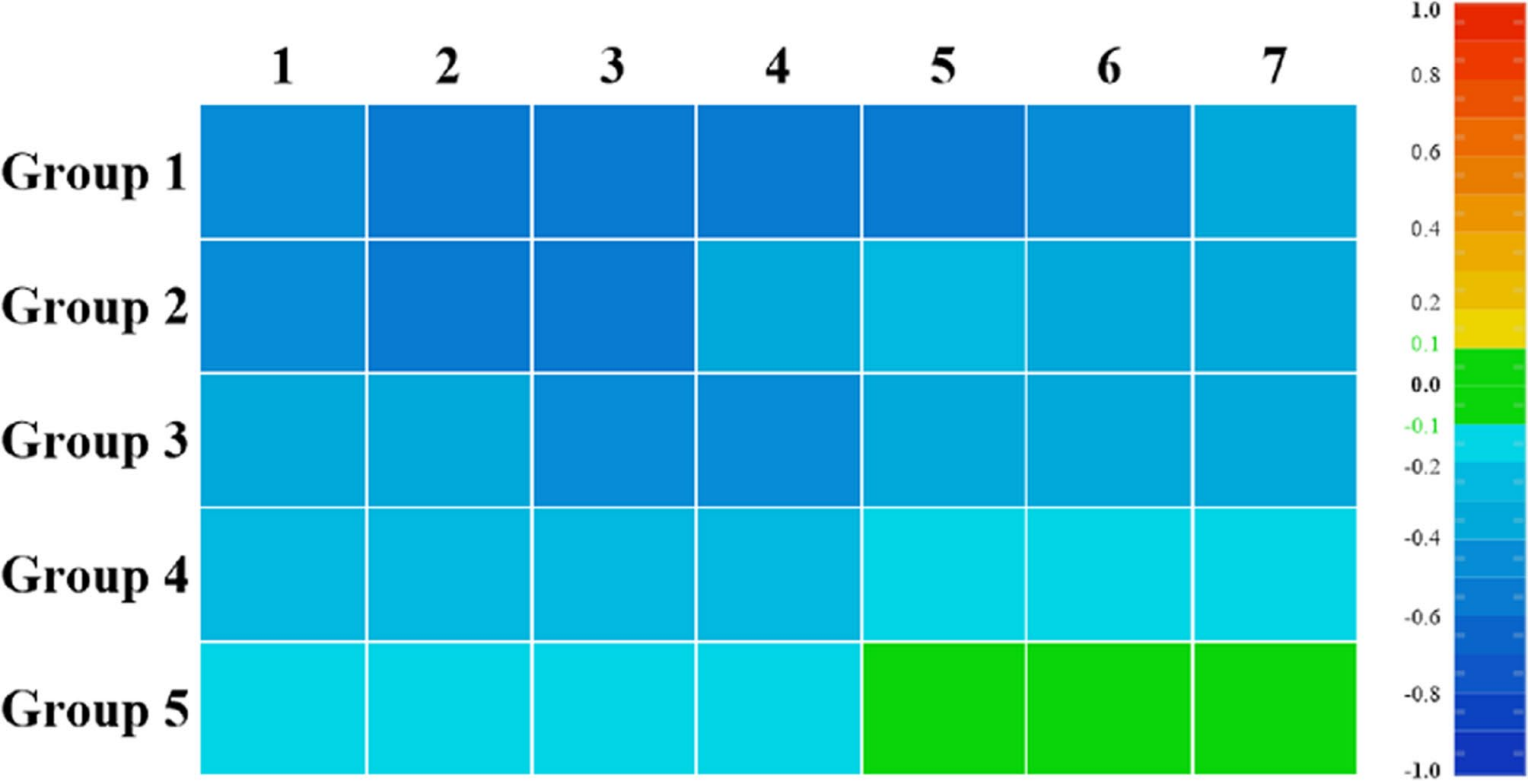
Heat maps of mean point deviations at the occlusal surfaces of different teeth

Analysis of the occlusal surfaces (Figs. 6 and 8; Table 3) revealed that the vertical height was consistently lower than designed (negative deviation) across most groups. In Group 1, extensive dark blue regions indicated pronounced vertical deficiencies, with the largest discrepancy at the canines (−0.60 ± 0.09 mm) and the smallest at the second molars (−0.33 ± 0.11 mm, Table 3). The magnitude of this negative deviation progressively decreased as the number of supporting teeth increased in the two-step protocol. A clear reduction in blue areas was observed from Group 1 to Group 5, with Group 5 showing predominantly green regions and minimal negative deviations (e.g., 0.19 ± 0.09 mm at canines to 0.09 ± 0.04 mm at second molars, Table 3). Localized yellow zones in the molar regions of Groups 4 and 5 (Fig. 6) suggest slight over-contouring where the test surfaces exceeded the reference.

**Table 3:** Comparison of point deviation measurements at the occlusal surfaces of different tooth position

	Tooth position							Statistics	
	Central incisors	Lateral incisors	Canines	First premolars	Second premolars	First molars	Second molars	F (6.63) *P*	η² 95% CI
Group 1	0.50 ± 0.08^Aa^	0.53 ± 0.11^Aa^	0.6 ± 0.09^Aa^	0.58 ± 0.12^Aa^	0.57 ± 0.15^Aa^	0.47 ± 0.08^Aab^	0.33 ± 0.11^Ab^	7.278 <0.001	0.409 0.280–0.336
Group 2	0.49 ± 0.10^Aabd^	0.51 ± 0.10^Aab^	0.57 ± 0.10^Aa^	0.39 ± 0.07^Bbd^	0.29 ± 0.02^Bc^	0.36 ± 0.06^Bcd^	0.39 ± 0.07^Abd^	14.308 < 0.001	0.577 0.249–0.283
Group 3	0.31 ± 0.05^Ba^	0.34 ± 0.06^Bac^	0.43 ± 0.04^Bb^	0.4 ± 0.03^Bbc^	0.31 ± 0.03^Ba^	0.33 ± 0.07^Bac^	0.33 ± 0.03^Aa^	9.899 < 0.001	0.485 0.189–0.237
Group 4	0.23 ± 0.03^Cab^	0.28 ± 0.04^Ba^	0.28 ± 0.05^Ca^	0.22 ± 0.07^Cab^	0.19 ± 0.05^Cb^	0.19 ± 0.09^Cab^	0.18 ± 0.08^Bab^	4.825 <0.001	0.315 0.163–0.210
Group 5	0.17 ± 0.07^Cab^	0.16 ± 0.07^Cab^	0.19 ± 0.09^Cb^	0.16 ± 0.09^Cab^	0.13 ± 0.07^Cab^	0.09 ± 0.04^Da^	0.09 ± 0.04^Ba^	3.511 0.005	0.251 0.143–0.175
F (4,45)	43.869	38.393	47.956	42.534	45.550	44.906	29.671		
*P*	< 0.001	< 0.001	< 0.001	< 0.001	< 0.001	< 0.001	< 0.001		
η²	0.796	0.774	0.810	0.791	0.802	0.800	0.725		
95% CI	0.298–0.383	0.320–0.410	0.363–0.463	0.304-0.400	0.250–0.347	0.244–0.330	0.224–0.299		

CI, confidence interval. Different uppercase letters indicate significant differences among groups within the same column (*P* < 0.05). Different lowercase letters indicate significant differences among groups within the same row (*P* < 0.05).

**Fig. 9:**
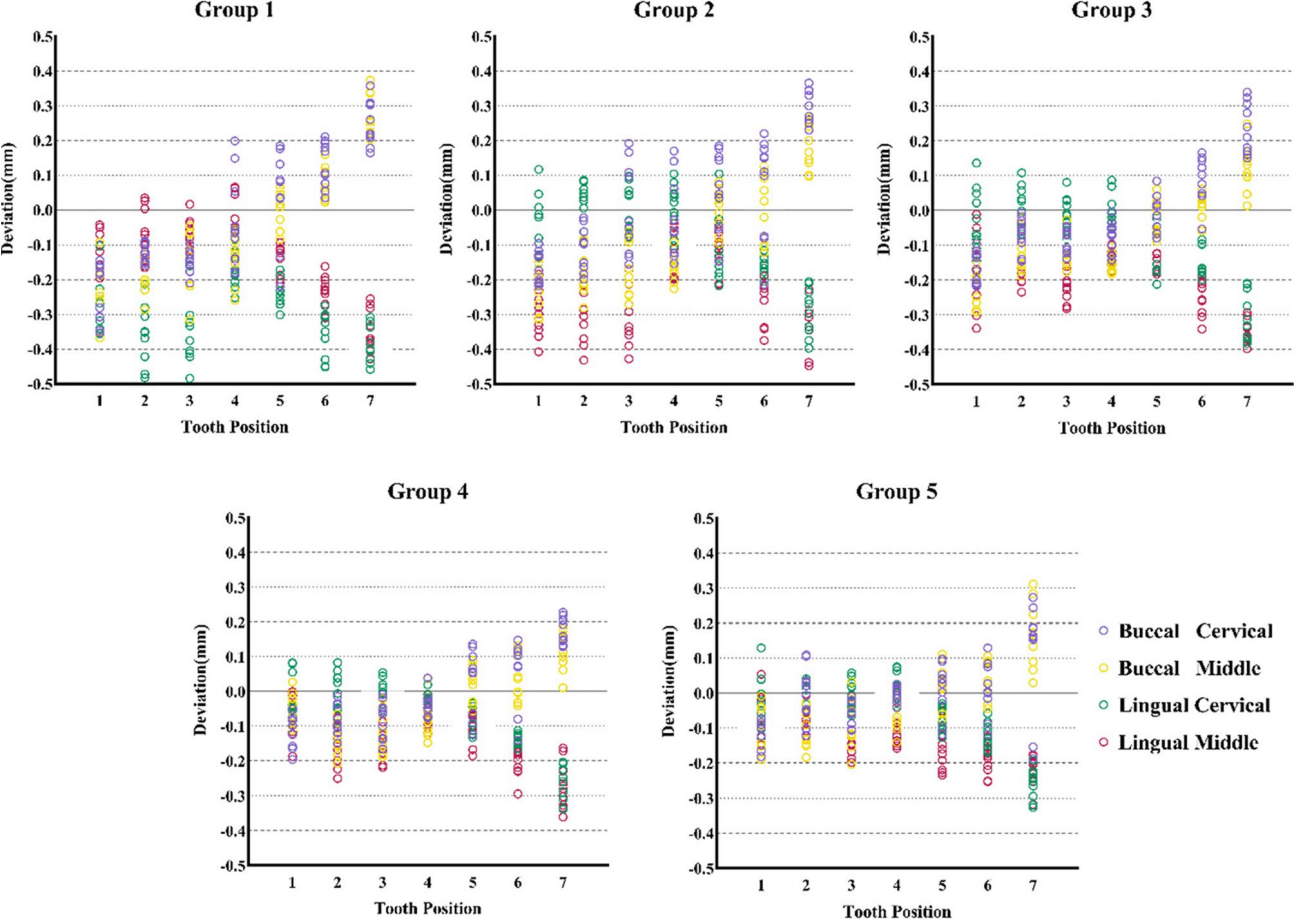
Scatter diagrams of point deviation measurements at the cervical and middle points of the buccal and lingual surfaces of different teeth position. Colored circles indicate measurement points: purple for buccal cervical, yellow for buccal middle, green for lingual cervical, and red for lingual middle

Measurements of buccal and lingual surface deviations are presented in scatter plots (Fig. 9). At the cervical and middle points, buccal surfaces showed a trend of thinning in the anterior teeth and thickening in the posterior teeth, with purple circles indicating cervical points and yellow circles indicating middle points. Lingual surfaces generally remained within the internal boundary of the design, where red circles correspond to middle points and green circles to cervical points. The smallest deviations were consistently observed at premolars, whereas the largest discrepancies occurred at second molars (*P* < 0.05). Cross-sectional images (Fig. 7) further showed that the distribution of stops affected thickness variations, particularly in the posterior regions.

## Discussion

This study demonstrates that two-step mock-up methods significantly enhance the trueness of complete-arch trial restorations compared to conventional one-step approaches, with trueness progressively improving as the number of supporting teeth increases. Therefore, the null hypothesis was rejected.

Although digitally fabricated trial restorations reduce chairside time and are suitable for no-prep rehabilitations [13–15], they present limitations such as challenging additive adjustments, bonding incompatibility with conventional resins, and technical sensitivity [11, 22–25]. In comparison, the conventional one-step silicone index mock-up method (Group 1) is widely used for its convenience. However, this study reveals its significant shortcomings. The lack of hard tissue stops combined with resin polymerization shrinkage resulted in substantial deviations [26, 27]. The greatest compression occurred in premolars closest to the pressure point, while second molars showed less reduction due to their free-end position and force distribution [28]. Despite variations among tooth positions, this method produced the largest 3D deviations among all groups, demonstrating unreliable trueness for complete-arch trial restorations (Table 1).

To address these issues, this study introduces a novel two-step mock-up method for complete-arch trial restorations. In the first step, unwaxed abutment teeth serve as hard-tissue reference zones; these areas then function as supports during the second transfer step. The results indicate that whole RMS trueness increases with the number of supporting teeth (Figs. 5 and 6; Table 1), likely due to the larger and more stable support area provided by the wider distribution of supported teeth [11]. Tooth-level 3D RMS deviations were smaller for teeth located closer to the supporting teeth (Fig. 5).

The observed negative deviations in occlusal height, in which trial restorations were consistently lower than the reference cast (Fig. 6-Fig. 8; Table 3), likely arise from the combined effects of silicone index compression under load and the inherent polymerization shrinkage of bis-acrylic resin (1–2%, according to manufacturer). Although more hard stops in Groups 2–5 reduced discrepancies [11], resin shrinkage remained a systematic error across all groups. The minimum occlusal deviation observed in Group 5 (Fig. 8, Table 3) indicates that even optimal support cannot fully compensate for material shrinkage. In the pits and fissures of posterior teeth, the limited flowability of silicone prevented full penetration, leading to higher bis-acrylic resin accumulation than intended. To control material-dependent confounders, resin injection, handling, and setting time were strictly performed within the manufacturer’s maximum recommended Limits, and environmental conditions were standardized. Since most of the resin shrinkage occurs within 10 min post-mixing, with significantly higher rates at 37 °C than 23 °C [29, 30], all procedures were conducted under controlled temperature (23 ± 1 °C) and humidity (50 ± 5%). To ensure dimensional stability, silicone indices were fabricated 24 h after wax-up printing, and trial restorations underwent 24-h stabilization before scanning.

The observed buccolingual deviations likely resulted from the U-shaped silicone index acting as a constrained mechanical system (Fig. 9) [31–33]. This mechanism is analogous to cantilever mechanics in implant prostheses, where the distal implant bears elevated stress, while the anterior portion of the silicone index acts as a fixed end, and the posterior free ends exhibit greater displacement upon loading [34, 35]. For anterior teeth, spatial and anatomical constraints (such as abutment size, orientation, and convergence angle) promoted uniform vertical sinking [36]. For posterior teeth, being more susceptible to deformation [37], the less constrained silicone deformed outward, particularly toward the buccal side where space allowed expansion, thereby increasing restoration thickness (purple and yellow circles, Fig. 9). Lingual deformation was limited by proximity to the opposing arch (green and red circles, Fig. 9). However, this remains a theoretical interpretation requiring future experimental validation.

In clinical practice, silicone indices are often manually fabricated and trimmed, leading to inconsistent margins due to operator variability [10]. To reduce this, a fan-shaped structure was added along the gingival margin in the diagnostic wax-up to standardize the shape and length of the silicone index margins, thereby promoting even resin distribution. Furthermore, manually made indices often have uneven thickness, which can cause unpredictable deformation under pressure. To address this, a custom 3D-printed mold was used to produce indices with uniform thickness (8 mm) [10, 18, 38], ensuring consistent mechanical strength and minimizing rigidity variations [38–41].

This study found that two-step methods produce trial restorations that more accurately replicate the wax-up than the one-step approach, which lacks defined stops during transfer, leading to unpredictable distortions such as reduced vertical height and buccolingual displacement. These inaccuracies may disrupt maxillomandibular relationships and require additional adjustments or remakes. In contrast, the two-step method with intraoral cross-mounting minimizes transfer errors and accurately captures occlusion, jaw relationship, and aesthetics. Once trial restorations are fixed in the first step, patients can better visualize and feel the proposed changes, allowing for systematic evaluation and adjustment of morphology, function, and occlusion, ultimately improving efficiency and patient satisfaction.

Several Limitations need consideration. First, only one silicone material and one provisional dental material were used, Limiting generalizability. Materials with different mechanical properties and flowability could affect the accuracy of trial restorations. Second, to control the magnitude and application points of the force, 500 g weights combined with bilateral premolar indicators were used [10]. However, this setup may not fully replicate dynamic clinical finger pressure. To address this limitation, programmable mechanical actuators could be employed in future studies to simulate dynamic finger force, thereby optimizing force distribution and improving clinical relevance. Additionally, this in vitro study used models from one patient, meaning it cannot fully replicate in vivo conditions (e.g., saliva, masticatory movements). Further research is needed to evaluate the two-step mock-up method’s applicability in varied intraoral scenarios and across different restoration types, as well as its performance with other materials in clinical practice.

## Conclusions

In this in vitro study, two-step mock-up methods significantly improved the trueness of complete-arch trial restorations compared to the one-step method. Increasing the number of supporting teeth in the intermediate waxing casts resulted in higher trueness. Further studies with different patients and materials are needed to validate these findings for clinical use.

## Data Availability

The datasets used and/or analyzed during the current study are not publicly available but are available from the corresponding author on reasonable request.

## Abbreviations

RMS: Root mean square
ANOVA: Analysis of variance
CAD: Computer-aided design
STL: Standard Tessellation Language
CI: Confidence Interval
3D: Three-dimensional
2D: Two-dimensional
